# Knowledge about non-invasive diagnostic tests for varices in liver cirrhosis: A questionnaire survey to the Gastroenterology Branch of the Liaoning Medical Association, China

**DOI:** 10.1093/gastro/gov031

**Published:** 2015-07-09

**Authors:** Xingshun Qi, Xiaozhong Guo, Hongyu Li, Xu Liu, Han Deng

**Affiliations:** Liver Cirrhosis Study Group, Department of Gastroenterology, General Hospital of Shenyang Military Area, Shenyang, Liaoning, China

**Keywords:** liver cirrhosis, varices, portal hypertension, non-invasive diagnostic tests, questionnaire survey

## Abstract

**Background and aims:** Due to the invasiveness of upper gastrointestinal endoscopy, non-invasive diagnostic tests for varices in liver cirrhosis have been widely established by numerous studies. A questionnaire survey, which was aimed at understanding the current knowledge about non-invasive diagnostic tests for varices in liver cirrhosis, was distributed among the members of Gastroenterology Branch of the Liaoning Medical Association.

**Methods:** A questionnaire assessing the knowledge about non-invasive diagnostic tests for varices was sent to 42 members who participated in the entire ninth committee. They were from 33 hospitals in 13 cities of Liaoning Province, China.

**Results:** Overall, 97.6% (41/42) of participants responded to the questionnaire. A majority of participants were chief physicians (85.4%), had >20 years of experience in clinical work of digestive diseases (80.5%) and worked at tertiary hospitals (97.6%). In 46.3% of participants’ departments, there were >200 patients with liver cirrhosis and gastroesophageal varices admitted every year. In 90.2% of participants’ departments, upper gastrointestinal endoscopy was regularly employed for the diagnosis of gastroesophageal varices. Only six (15%) participants often used non-invasive diagnostic tests for varices in clinical practice. Thirty (75%) participants knew at least one non-invasive diagnostic test for varices. The knowledge of at least one non-invasive diagnostic test was significantly associated with the number of cirrhotic patients with varices (*P* = 0.038) or the regular use of gastrointestinal endoscopy to diagnose varices (*P* = 0.022).

**Conclusions:** This questionnaire survey suggested that non-invasive diagnostic tests for varices in liver cirrhosis were rarely or never used in clinical practices in Liaoning Province, China. Reliable, non-invasive indexes should be further explored in a well-designed multi-center observational study.

## Introduction

**Figure 1.**
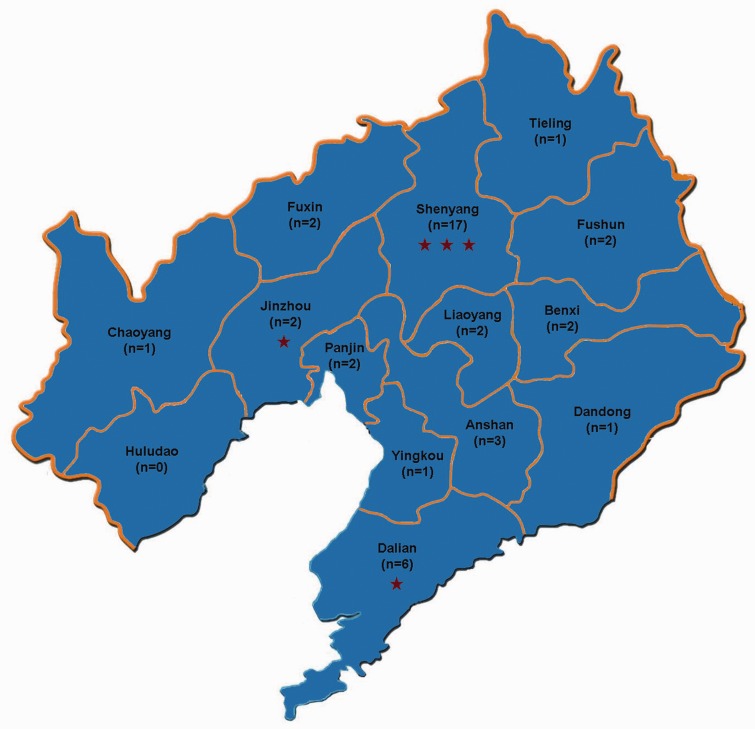
Map of Liaoning Province with 14 cities. Note: The number of members from different cities who participated in the questionnaire survey is recorded in parentheses. Asterisk represents a major medical university or college in different cities.

Current practice guidelines recommend that the presence and severity of gastroesophageal varices be screened by upper gastrointestinal endoscopy at the first diagnosis of liver cirrhosis [1,2]. The use of primary prophylaxis is largely dependent upon the grade of varix. For cirrhotic patients with no or small varices, non-selective beta-blockers should not be recommended [3,4]. For cirrhotic patients with medium to large varices, non-selective beta-blockers or variceal band ligation should be initiated to decrease the incidence of first variceal bleeding as soon as possible [5–7]. Due to the invasiveness of upper gastrointestinal endoscopy [8,9], numerous non-invasive diagnostic tests for varices have been explored such as aspartate aminotransferase (AST)-to-platelets, (PLT) ratio index (APRI), PLT-to-spleen diameter ratio (PSR), liver and spleen stiffness measurement by Fibroscan, FIB-4 index and AST-to-alanine aminotransferase (ALT) ratio (AAR), etc [10,11]. However, it remains unclear whether or not these non-invasive diagnostic tests have been used in actual clinical practice. Herein, we designed a questionnaire survey to understand the current knowledge about non-invasive diagnostic tests for varices in liver cirrhosis in Liaoning Province. Notably, in this province, there are 14 cities and 5 major universities or colleges of clinical medicine (e.g. Chinese Medical University, Liaoning Medical University, Liaoning University of Chinese Traditional Medicine, Dalian Medical University and Shenyang Medical College) (Figure 1).

## Methods

### Study design

A questionnaire survey was conducted on May 8, 2015, among the ninth entire committee members of the Gastroenterology Branch of the Liaoning Medical Association. This committee is composed of 53 members from 42 hospitals in 14 cities of Liaoning Province. Among them, 42 members from 33 hospitals in 13 cities participated in this survey (Figure 1).

First, the participants were informed about the objective of this questionnaire survey. Notably, all of them did not know about this pre-planned survey except for the corresponding author of this paper (who is the chairman of the committee and the guarantee of this work (XG)),. They were asked to carefully and faithfully complete this questionnaire survey. Second, they filled out their personal information including name, sex, age, position, city, affiliation and email address. Third, they replied to 20 questions. These questions could be divided into 4 major sections: (1) participants and hospitals, (2) patients with liver cirrhosis and gastroesophageal varices, (3) upper gastrointestinal endoscopy and (4) non-invasive diagnostic tests. This questionnaire was translated by XQ into the English language in Table 1.

**Table 1.** Questionnaire

1) What is your title?
A. Chief physician. B. Vice-chief physician. C. Attending physician. D. Other.
2) How long have you participated in the clinical work of digestive diseases?
A. >20 years. B. 10–20 years. C. <10 years. D. Other.
3) What is the grade of your hospital?
A. Tertiary, class A. B. Tertiary, class B. C. Second level, class A. D. Other.
4) How many beds does your hospital have?
A. >1000. B. 800–1000. C. 500–800. D. Other.
5) How many beds does the Department of Gastroenterology have in your hospital?
A. >120. B. 80–120. C. 40–80. D. Other.
6) How many patients with liver cirrhosis are admitted to the Department of Gastroenterology of your hospital every year?
A. >200. B. 100–200. C. 50–100. D. Other.
7) Is upper gastrointestinal endoscopy performed at the Department of Gastroenterology of your hospital?
A. Yes. B. No.
8) How many patients with liver cirrhosis are diagnosed with gastroesophageal varices on upper gastrointestinal endoscopy at the Department of Gastroenterology of your hospital every year?
A. >200. B. 100–200. C. 50–100. D. Other.
9) Is upper gastrointestinal endoscopy regularly performed to diagnose gastroesophageal varices at the Department of Gastroenterology of your hospital every year?
A. Yes. B. No.
10) If the answer to the ninth question is ‘no’, please specify the reasons.
A. Upper gastrointestinal endoscopy may increase the risk of bleeding. B. Upper gastrointestinal endoscopy is unnecessary. C. Other.
11) Is endoscopic treatment is employed for the management of gastroesophageal varices at the Department of Gastroenterology of your hospital?
A. Yes. B. No.
12) Is endoscopic variceal band ligation employed for the management of gastroesophageal varices at the Department of Gastroenterology of your hospital?
A. Yes. B. No.
13) Is endoscopic sclerotherapy employed for the management of gastroesophageal varices at the Department of Gastroenterology of your hospital?
A. Yes. B. No.
14) Is endoscopic tissue adhesive injection employed for the management of gastroesophageal varices at the Department of Gastroenterology of your hospital?
A. Yes. B. No.
15) Do you know about the methods for the non-invasive diagnosis of varices in liver cirrhosis?
A. Familiar, often used. B. Acquainted, rarely used. C. Acquainted, never used. D. Never heard.
16) Which of the following non-invasive diagnostic tests for varices do you know?
A. APRI (AST and PLT). B. PSR (PLT and diameter of spleen). C. Fibroscan (liver and spleen stiffness). D. FIB-4 (age, AST, ALTand PLT). E. AAR (AST and ALT). F. Other.
17) Do you use the non-invasive diagnostic tests for varices in clinical practice?
A. Yes. B. No.
18) What do you think about the reliability of non-invasive diagnostic tests for varices in clinical practice?
A. Very reliable. B. Some reference values. C. Unreliable.
19) Have you published any papers about the role of non-invasive diagnostic tests for varices?
A. Yes. B. No.
20) Would you like to participate in the multicenter observational study to establish a reliable non-invasive index for the diagnosis of varices in future?
A. Yes. B. No.

### Statistical analysis

All statistical analyses were performed using SPSS Statistics 17.0.0. Continuous and categorical data were expressed as mean with standard error and frequency with percentage, respectively. Categorical data among groups were compared by using Pearson’s chi-square test. Pie charts were also drawn to clearly show the main results proportionally. Two-sided *P* < 0.05 was considered as statistically significant.

## Results

### Questionnaire

Overall, 41 of the 42 participants responded to the questionnaire. One participant, who did not respond to the questionnaire, mentioned that he and his colleague had the same answers. Thirty-eight participants answered all 20 questions. Three participants answered 19 of the 20 questions, because two participants did not answer the 18th question regarding the reliability of non-invasive diagnostic tests for varices, and one participant did not answer the 15th question regarding the knowledge of non-invasive diagnostic tests of varices. In addition, the answer to the 18th question regarding reliability of non-invasive diagnostic tests for varices was ineffective for two participants because they chose ‘D’. The answer to the 19th question regarding publication of non-invasive diagnostic tests for varices was ineffective in one participant because he chose ‘D’. Thus, there were 40 effective answers for the 15th question, 37 effective answers for the 18th question and 40 effective answers for the 19th question.

### Participants

The basic information about the participants and their hospitals and departments is summarized in Table 2. A majority of participants were chief physicians (85.4%), had >20 year of experience in the clinical work of digestive diseases (80.5%) and worked at tertiary hospitals (97.6%). The number of beds was >120 in 19.5% of participants’ departments, 80–120 in 24.4% of them and 40–80 in 36.6% of them. The number of patients with liver cirrhosis admitted every year was >200 in 46.3% of participants’ departments. The number of patients with liver cirrhosis and gastroesophageal varices every year was >200 in 46.3% of participants’ departments.

**Table 2.** Basic information of participants and their hospitals and departments

Variables	Results
Male, n (%)	20 (48.8)
Age, years	49.39 ± 0.82
Position, n (%)	
President of hospital	1 (2.4)
Vice president of hospital	2 (4.9)
Director of department	30 (73.2)
Vice director of department	7 (17.1)
No position	1 (2.4)
Title, n (%)	
Chief physician	35 (85.4)
Vice-chief physician	6 (14.6)
Experience in the clinical work of digestive diseases, n (%)	
>20 years	33 (80.5)
10–20 years	8 (19.5)
Grade of hospital, n (%)	
Tertiary, class A	35 (85.4)
Tertiary, class B	5 (12.2)
Second level, class A	1 (2.4)
Number of beds in the hospital, n (%)	
>1000	34 (82.9)
800–1000	3 (7.3)
500–800	3 (7.3)
Others	1 (2.4)
Number of beds in the Department of Gastroenterology, n (%)	
>120	8 (19.5)
80–120	10 (24.4)
40–80	15 (36.6)
Others	8 (19.5)
Number of patients with liver cirrhosis admitted to the Department of Gastroenterology every year, n (%)	
>200	19 (46.3)
100–200	9 (22.0)
50–100	13 (31.7)
Number of patients with liver cirrhosis and gastroesophageal varices admitted to the Department of Gastroenterology every year, n (%)	
>200	15 (36.6)
100–200	7 (17.1)
50–100	16 (39.0)
Others	3 (7.3)

### Upper gastrointestinal endoscopy

Regarding the seventh question, nearly all participants’ departments had upper gastrointestinal endoscopy facilities (97.6%, 39/41). Regarding the ninth question, upper gastrointestinal endoscopy was regularly employed for diagnosing gastroesophageal varices in 90.2% (37/41) of participants’ departments. In the remaining four participants’ departments, upper gastrointestinal endoscopy was not employed because they were worried about an increased risk of bleeding with upper gastrointestinal endoscopy (n = 3) or thought that upper gastrointestinal endoscopy was unnecessary (n = 1). Notably, in one participant, the answer to the seventh question was ‘B’, but that to the ninth question was ‘A’. Finally, there were 36 participants’ departments with upper gastrointestinal endoscopy facilities and use of upper gastrointestinal endoscopy to diagnose varices in liver cirrhosis.

Regarding the 11th question, upper gastrointestinal endoscopy was employed for the management of gastroesophageal varices in 80.5% (33/41) of participants’ departments. In details, endoscopic variceal band ligation, sclerotherapy and tissue adhesive injection were employed in 19 (46.3%), 24 (58.5%) and 15 (36.6%) participants’ departments, respectively.

#### Non-invasive diagnosis of varices in liver cirrhosis

**Figure 2.**
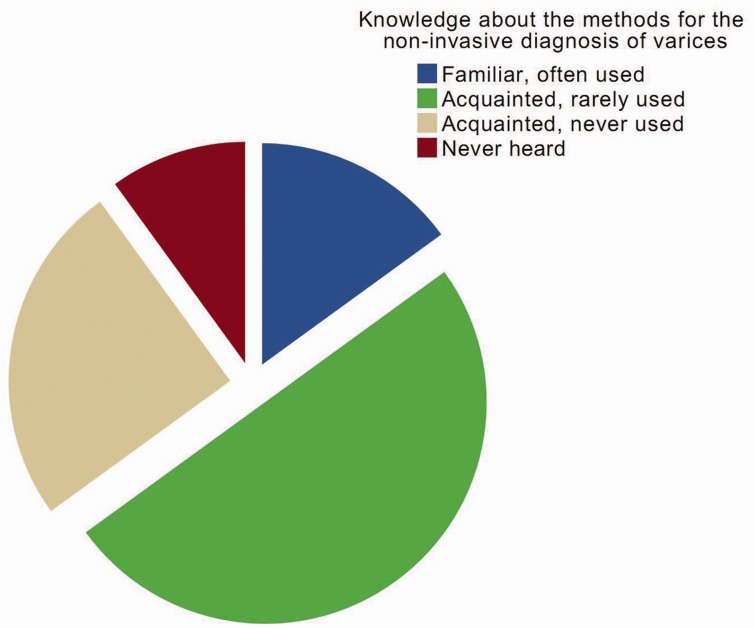
Knowledge about the non-invasive diagnostic tests of varices according to the participant’s familiarity.

Regarding the 15th question, only six participants were familiar with and often used non-invasive diagnostic tests for varices (15%, 6/40). By comparison, most participants were acquainted with non-invasive diagnostic tests, but rarely or never used them in clinical practice (75%, 30/40). Four participants had never heard of them (Figure 2).

**Figure 3.**
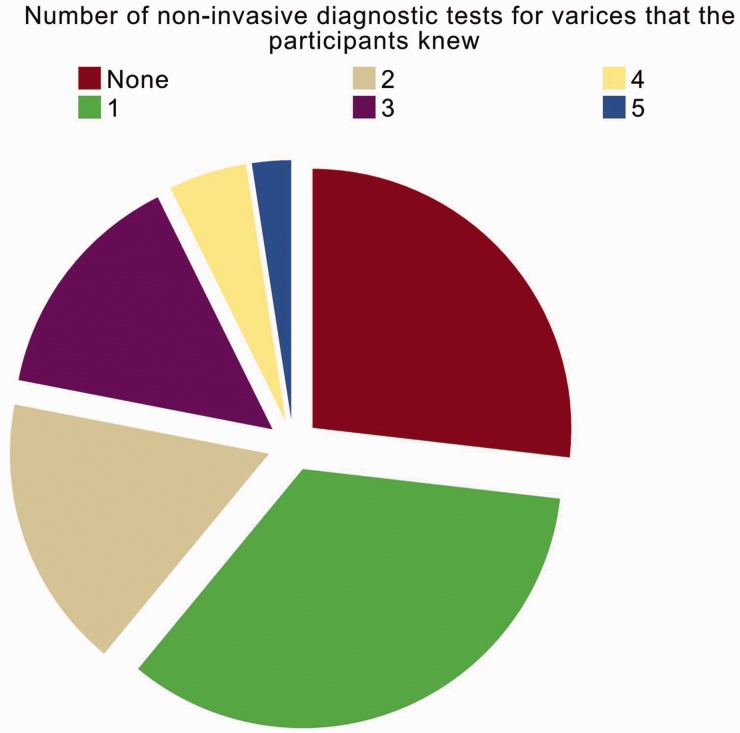
Knowledge about the non-invasive diagnostic tests of varices according to the number of non-invasive methods that the participant knew.

**Figure 4.**
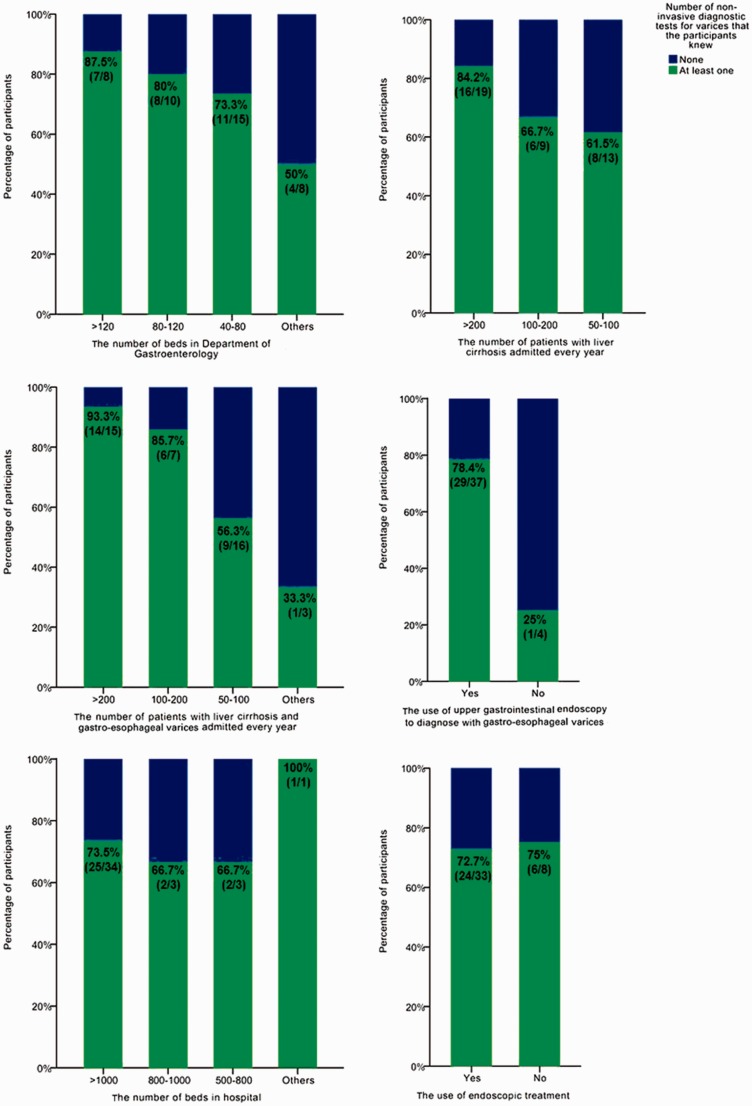
Factors associated with the proportion of participants who knew at least one non-invasive diagnostic test of varices.

Regarding the 16th question, 30 participants knew at least one non-invasive diagnostic test for varices (73.2%, 30/41) (Figure 3). The proportion of participants who knew at least one non-invasive diagnostic test for varices was significantly associated with the number of cirrhotic patients with varices (*P* = 0.038) and the regular use of gastrointestinal endoscopy to diagnose varices (*P* = 0.022) but not with the number of beds in the participants’ departments (*P* = 0.353) or the number of cirrhotic patients (*P* = 0.321). In addition, the proportion might not be associated with the number of beds in the participants’ hospital or the use of endoscopic treatment (Figure 4).

In detail, nine (22.0%), 19 (46.3%), 18 (43.9%), nine (22.0%) and four (9.8%) participants knew about APRI, PSR, liver and/or spleen stiffness measurement by Fibroscan, FIB-4 and AAR for non-invasive diagnosis of varices, respectively. However, only nine (22.0%) participants used these non-invasive diagnostic tests. Non-invasive diagnostic tests for varices were considered reliable, limited and unreliable in 4, 31 and 2 of the 37 participants with effective answers to the 18th question.

Regarding the 19th question, only two participants had published papers related to non-invasive diagnosis of varices. Regarding the 20th question, all participants were willing to participate in a multi-center observational study in the future.

## Discussion

To our knowledge, this questionnaire survey for the first time explored knowledge about non-invasive diagnosis of varices in liver cirrhosis with a medical society of digestive diseases. An important finding of our survey was that few participants often used non-invasive diagnostic tests for varices in clinical practice. This might be explained by the current status in this field. Upper gastrointestinal endoscopy remains the gold standard test for the diagnosis of varices. Until now, no reliable non-invasive indexes have been established yet. Thus, the physicians hesitate to accept the concept of non-invasive diagnosis of varices. To further promote the relevant knowledge, more valid non-invasive scores should be produced, more education should be provided, and more clinical research should be actively launched.

Another preliminary finding was that 73.2% of participants knew at least one non-invasive diagnostic test for varices. The knowledge about non-invasive diagnosis of varices might be influenced by their experiences managing liver cirrhosis and varices, such as the number of cirrhotic patients with varices admitted and whether or not gastrointestinal endoscopy was regularly used to diagnose varices.

The most common non-invasive index that the participants knew was PSR, followed by liver and/or spleen stiffness measurement by Fibroscan, APRI, FIB-4 and AAR. Indeed, based on a recent meta-analysis, the role of PSR for the non-invasive diagnosis of varices has been evaluated by at least 20 studies involving 3063 patients [12]. More importantly, PSR had a good summary sensitivity and specificity of 0.92 and 0.87, respectively. Additionally, the role of spleen stiffness measurement for the non-invasive diagnosis of varices has been evaluated in at least 12 studies. It has a relatively good sensitivity and specificity of 78% and 76%, respectively [13]. Thus, it was readily understood that more physicians knew PSR and spleen stiffness measurement for non-invasive diagnosis of varices in liver cirrhosis. Recently, our unpublished study has systematically evaluated the diagnostic accuracy of APRI, FIB-4 and AAR for the prediction of varices in liver cirrhosis. However, their diagnostic accuracy was relatively modest (PROSPERO registration number: CRD42015017519). Additionally, we also retrospectively evaluated the role of four major serum liver fibrosis markers in diagnosing the presence of varices in 118 patients [14]. But the results were disappointing, such that none of them could be recommended in clinical practice.

On the other hand, the results of this questionnaire survey are necessary for initiating a multi-center observational study to establish a useful and reliable index for non-invasive diagnosis of varices. Ideally, the future study should be composed of the training and validation sets. In the training set, a non-invasive index should be produced according to the data collected from the hospitals of Liaoning provinces during a relatively earlier period. In the internal and/or external validation set, the diagnostic accuracy of this index should be confirmed. Notably, the candidates for such a study should meet two major criteria: (1) the departments with upper gastrointestinal endoscopy (i.e. seventh question) and (2) the regular use of endoscopy to diagnose varices in liver cirrhosis (i.e. ninth question). Accordingly, a total of 36 participants from 28 hospitals met the two major criteria. Certainly, the optimal candidates should also meet another three minor criteria: (1) a ample experience in the clinical work of digestive diseases (i.e. second question); (2) more cirrhotic patients with varices admitted to their departments every year (i.e. nineth question); and (3) the use of endoscopic management for varices and variceal bleeding (i.e. 11th–14th questions).

Several limitations should be also clarified. First, the number of participants was relatively small. This was primarily attributed to the fact that this questionnaire was sent only to members of the Gastroenterology Branch of the Liaoning Medical Association. Certainly, it should be noted that their knowledge could accurately reflect the current status of Liaoning Province. Second, not all members participated in the survey (79%, 42/53). Third, this questionnaire survey was limited to Liaoning Province, China. Thus, we had to acknowledge that our findings might not be generalized to the other societies of gastroenterology.

In conclusion, non-invasive diagnostic tests for varices in liver cirrhosis were rarely or never used in clinical practice. Reliable, non-invasive indexes should be further established in a well-designed, multicenter observational study. Additionally, based on the results of a questionnaire survey, the candidates for such a study can be identified.
